# Nephrological disorders and neurological involvement in pediatric primary Sjogren syndrome:a case report and review of literature

**DOI:** 10.1186/s12969-020-00431-y

**Published:** 2020-05-24

**Authors:** Jingya Zhao, Qin Chen, Yunyun Zhu, Meng Zhao, Jun Liu, Zhenzhong Zhang, Xiaoting Gong

**Affiliations:** 1 Department of Nephrology, Tongde Hospital of Zhejiang Province, Gucui Road, No.234, Hangzhou, 310012 People’s Republic of China; 2 Department of Pathology, Tongde Hospital of Zhejiang Province, Hangzhou, People’s Republic of China; 3 Department of Laboratory, Tongde Hospital of Zhejiang Province, Hangzhou, People’s Republic of China

**Keywords:** Neurologic manifestations, Kidney diseases, Sjogren’s syndrome, Children

## Abstract

**Background:**

Sjögren syndrome (SS) is a rare disease in pediatrics, and little attention has been paid to the clinical feature in these patients. To date, there are few cases concern about neurological and nephrological disorders in childhood Sjögren syndrome. We describe a case of Sjögren syndrome in a 12-year-old girl who developed neurological disorders and interstitial nephritis and review the literature currently available on this topic.

**Case presentation:**

A 12-year-old girl was admitted to our hospital for arthritis and glucosuria. She was required to do labial gland and renal biopsy, because the positive for anti-nuclear antibody and anti-Sjögren syndrome B (anti-SSB) antibody. Then the biopsy was performed revealing the lymphocytic infiltrate in the small area and renal tubular interstitial damage,thus the diagnosis of Sjögren syndrome with tubular interstitial damage was made. Three months later, she presented again with headache, fever, nausea, vomiting and was recovered without drug therapy. Based on the patient’s medical history, laboratory and imaging examination, and treatment, we speculate that the disorders of the nervous system were caused by the Sjögren syndrome. The girl has stable renal function and no residual nervous system damage in the next 1.5 years, but she underwent low dose prednisone therapy because of persistent renal glucosuria.

**Conclusions:**

Nephrological disorders and neurological involvement are rare manifestations of Sjögren syndrome in children, and rarely presented as the initial symptoms. It should be suspected in children presenting with unexplained renal diseases, neurological abnormalities, or unexplained fever. Although there is no guidelines on the diagnosis and treatment of children Sjögren syndrome are currently available, early recognition and the appropriate treatment of renal damage and neurologic involvement would improve prognosis and prevent complications.

## Background

Sjögren syndrome (SS) is a complex autoimmune disease characterized by inflammation of the lacrimal and salivary glands leading to keratoconjunctivitis sicca and xerostomia with up to half of affected adults developing additional extraglandular manifestations. It is one of the most common autoimmune rheumatic diseases in adults, and organ systems associated with extraglandular manifestations (EGMs) are frequently involved, including the nervous, pulmonary, and vascular system [1]. However, the incidence of primary SS in children is much lower than in adults, and the presentation of childhood Sjögren syndrome often differs between children and adults. To date, few cases of primary childhood SS combined with neurological disorders and nephrological damage have been reported in the literature. Here, we describe a case of juvenile Sjögren syndrome in a 12-year-old girl who developed arthralgia, neurological disorders, and interstitial nephritis.

## Case presentation

**Fig. 1:**
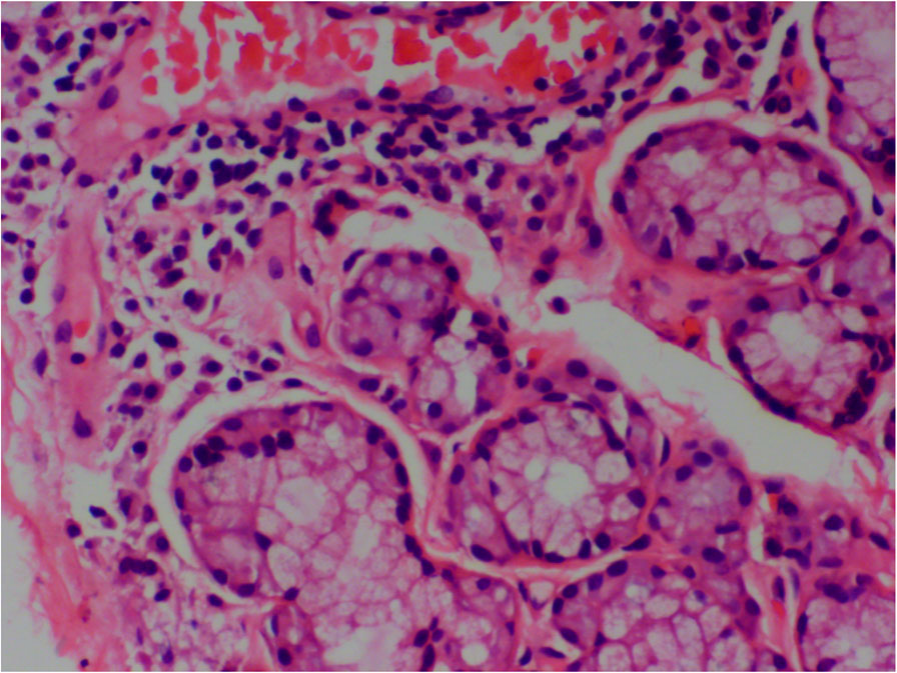
minor salivary gland biospy

**Fig. 2:**
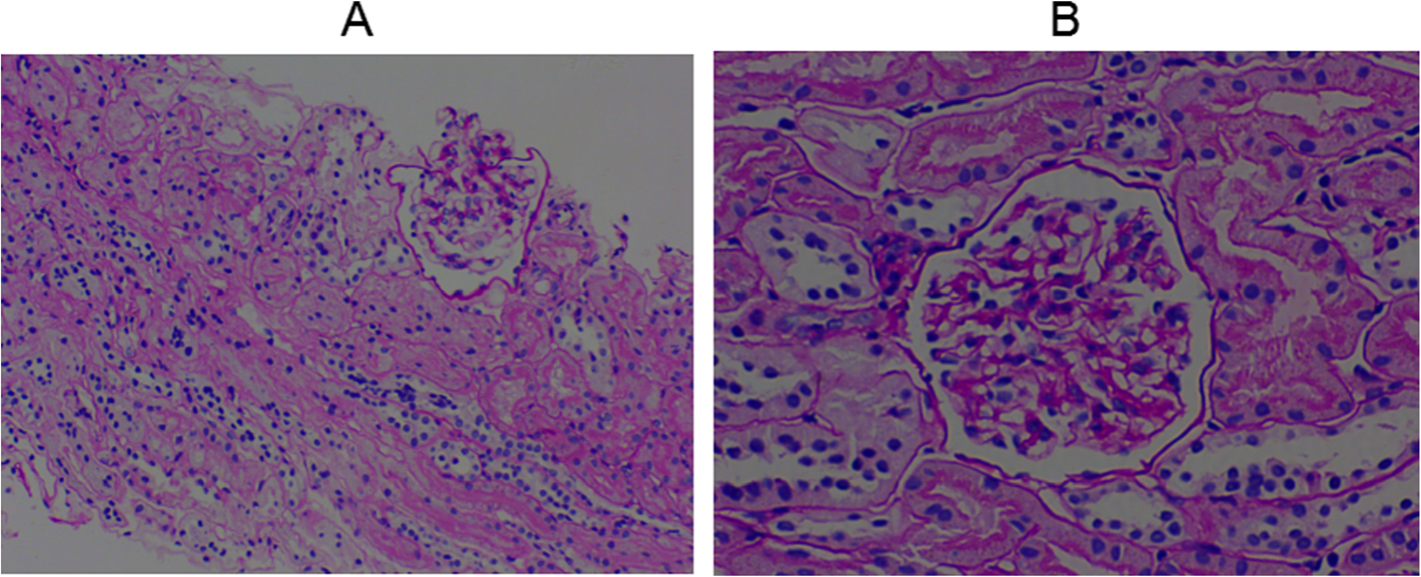
Kidney biopsy specimen

**Fig. 3:**
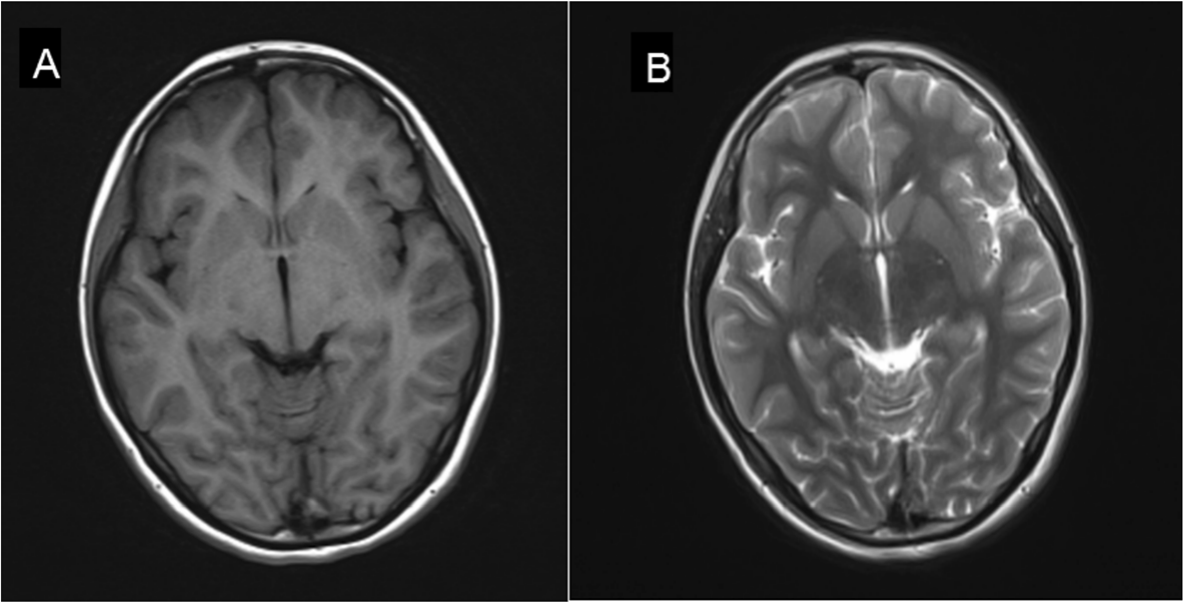
T1-weighted and T2-weighted image showing normal signal intensity in the parenchymal and cerebellum. No abnormally was found in the shape, size and position of ventricle, cistern and sulci

A 12-year-old girl presented to our clinic with left knee joint pain that had lasted for 7 days. The patient had no symptoms of fever, skin purpura, dry mouth, dry eyes, or parotitis. There was no past history of neck or face radiotherapy. Her physical examination revealed swelling in the left knee joint. Other physical findings were normal. Laboratory testing indicated normal complete blood counts and an erythrocyte sedimentation rate (ESR) of 33 mm (0–20 mm/h). The patient tested positive for glucosuria upon urine screening, but was negative for hematuria and proteinuria. Then, we administered an oral glucose tolerance test (OGTT) and measured glycosylated hemoglobin A-1c(HbA1c) to evaluate glucose intolerance. Eventually,she was diagnosed as having renal glucosuria (RG) with normal glucose tolerance and normal HbA1c. She also tested positive for anti-nuclear antibody (ANA) (1:100), anti-Sjögren syndrome B (anti-SSB/Ro), Perinuclear antineutrophil cytoplasmic antibody(P-ANCA)(1:10), and ESR (33 mm/h). Tests for anti-Sjögren syndrome A (anti-SSA/La), anti-Sm, anti-double stranded DNA (anti-dsDNA), anti-ribonnucleoprotein (anti-RNP), ani-Scl70, anti-phospholipid, anti-Jo-1, anti-myeloperoxidase antineutrophil cytoplasmic antibody (anti-MPO-ANCA), rheumatoid factor (RF), anti-cyclic citrullinated peptide (anti-CCP) antibody, hepatitis B virus (HBV), hepatitis C virus (HCV), human immunodeficiency virus (HIV), IgG, IgA, IgM, C3, and C4 were negative or normal. Ophthalmological assessment revealed her schirmer’s test was positive. Ultrasonography of shallow lymph glands showed enlarged lymph nodes in the bilateral parotid gland. Histopathological analysis of minor salivary gland biopsy showed a lymphocytic infiltrate around the ducts and acinus in the small area (> 50 lymphocytes/4 mm^2^; Fig. 1). Renal biopsy showed tubular interstitial damage (Fig. 2). Light microscopy demonstrated previously unapparent proliferation of glomerular mesangial cells, increases in mesangial matrix, tubular interstitial and acinus damage, renal tubule epithelial cell swelling and degeneration, focal tubular atrophy, and tubular epithelial cell fusion (< 5%), while an interstitial lymphoplasmacytic infiltrate was not obvious. Electron microscopy revealed partial foot process fusion and no other significant ultrastructural abnormalities. Immunofluorescence showed no deposition of immunoglobulins (IgG, IgA, and IgM) or complement (C3, C4, and C1q), and the kappa and lambda chains were also negative. Based on the 2012 American College of Rheumatology Classification (ACR) criteria for Sjögren syndrome and kidney biopsy, she was diagnosed as primary Sjogren syndrome (pSS) with tubular interstitial damage. Her treatment consisting of celebrex (200 mg/d) and hydroxychloroquine (100 mg/d) was administered during the first week, with hydroxychloroquine (200 mg/d) and sulfasalazine enteric-coated tablets (400 mg/d) during the next half year of treatment. After the first 2 months of treatment, the girl’s joint pain was in complete remission and the complete blood counts and erythrocyte sedimentation rate were all normal. Three months later, she developed temporal headache, which was intermittent,being worst in the afternoon and relieved in the morning. The next day, she began to have a fever of 38.8 °C with nausea and vomiting. With worsening symptoms, the patient came to our hospital directly, where an MRI scan of the brain was normal (Fig. 3). Her physical examination showed superficial sensation disorder in the upper limbs and Kernig signs. Complete blood counts,c-reactive protein, procalcitonin, and the erythrocyte sedimentation rate were all unremarkable. For further investigation, lumbar puncture was performed, which revealed normal cerebrospinal fluid (CSF) pressure (> 125 mmH_2_O). High leucocyte counts of 30 cells 10^6 L with lymphocytic predominance (60%),a CSF glucose level of 3.1 mmol/L,a chlorine level of 126.1 mmol/L, and a CSF protein level of 206 mg/L were noted. Cytology of the CSF showed large lymphocytes and occasional histiocytic cells with normal morphology. Bacterial culture of the CSF was negative. Virus antibody testing of the CSF was negative for herpes simplex 1 and 2, cytomegalovirus, echovirus, coxsackievirus, and Epstein-Barr virus. *Cryptococcus neoformans* and *Mycobacterium tuberculosis* were also not found in the CSF. After these test results, she was diagnosed with aseptic meningoencephalitis but we could not exclude the possibility of viral meningitis. Therefore, the patient was treated with intravenous acyclovir. However,due to drug allergy,we stopped acyclovir treatment early. After 3 days, her headache and rash were significantly relieved. Based on the patient’s medical history, CSF examination, and treatment, we speculate that the disorders of the nervous system were more likely caused by the pSS. During the follow up of 1.5 years, her renal function was stable and no residual nervous system damage was apparent. She underwent low dose prednisone therapy (5-10 mg/d) for half a year because of persistent renal glucosuria.

## Discussion and conclusions

We aimed to review all full-text, peer-review publications reporting childhood Sjogren syndrome with kidney or nerve damage. Records were identified from the PubMed, EMBASE databases. The search terms were primary Sjogren syndrome, child, children, and childhood. Results were limited to case reports written in English. The search date was December 23, 2019.

**Fig. 4:**
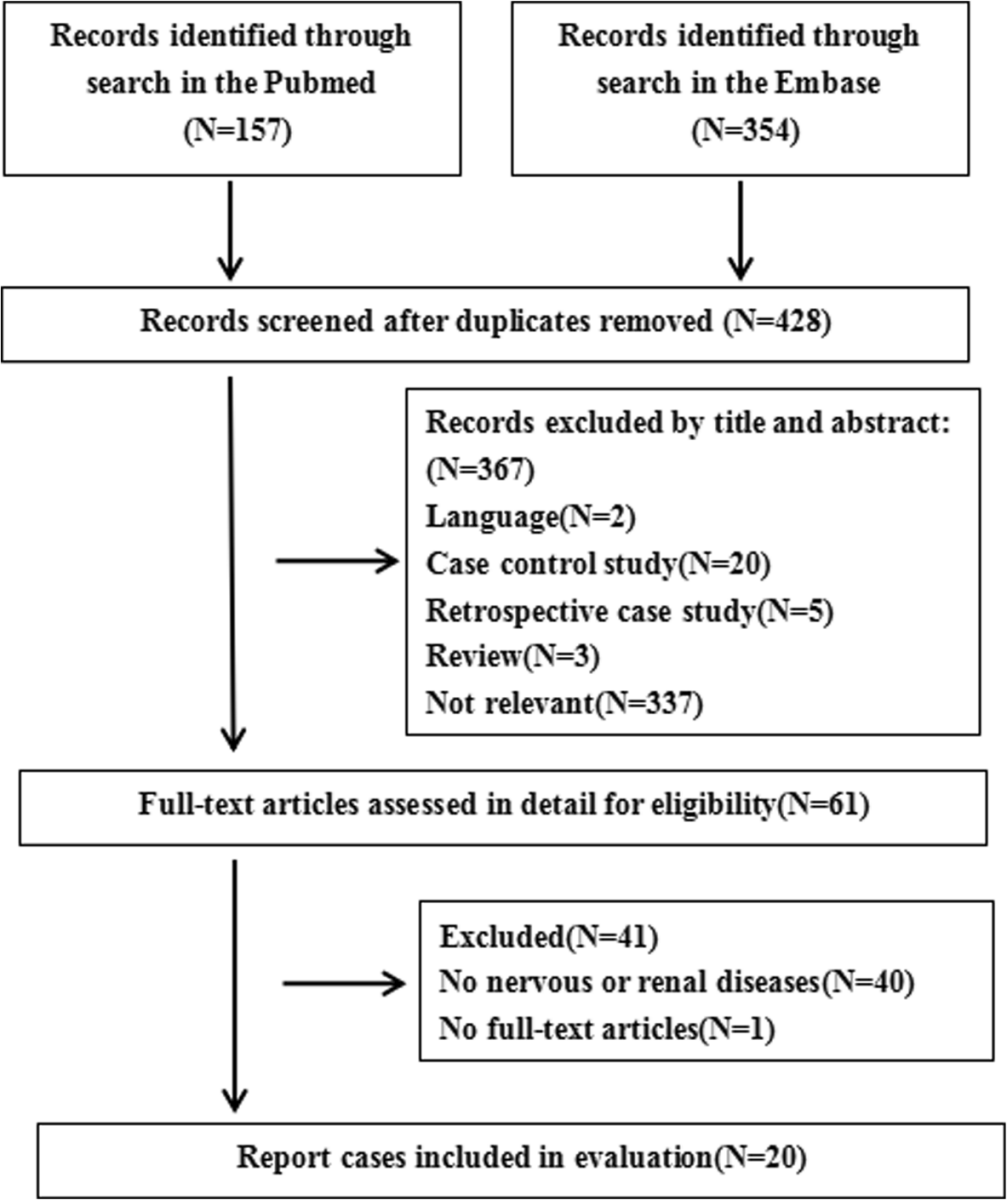
Study selection flow chart

The initial search yielded 511 articles, after excluding the duplicate articles and reading titles and abstracts, 61 papers were then read in detail. Finally, 20 case reports were included in the literature review after extracting and analyzing the data from the articles (Fig. 4). The information that was extracted from the papers were as follows: references and year, age and gender of patient, symptoms at onset, dry eyes or mouth, parotitis,neurologic manifestation, renal damage, elevated ANA, presence of anti-SSA and SSB antibodies, ESR, RF, hyperglobulinemic, schirmer test, CSF, renal and salivary gland biopsy and immunomodulatory therapy (Table 1) [2–21].

**Table 1:** neurological and nephrological manifestation in childhood Sjogren syndrome

Case	1	2	3	4	5	6	7	8	9	10	11
References/Year	Matsui [2]	Kornitzer [3]	Arabshahi [4]	Bogdanovic [5]	Kumon [6]	Pessler [7]	Jung [8]	Igarashi [9]	Gottfried [10]	Wong [11]	Kagan [12]
Age (Gender)	9/F	6/F	11/F	13/F	14/F	6/F	11/F	12/F	9/F	16/F	12/F
Symptoms at onset	fever	fever headache	optic neuritis	nephrocalcinosis	fever arthralgia	parotitis	hematuria proteinuria	parotitis	fever conjunctivitis	headache	AKI
Dry eyes or mouth	NM	N	NM	NM	NM	NM	Y	Y	Y	N	NM
Parotitis	NM	NM	NM	Y	N	NM	N	Y	Y	Y	Y
Neurologic manifestation	mental disorder gaze deviation ambulation difficulty	mental disorder muscle weakness	muscle weakness urinary incontinence	N	arm numbness hypalgesia thermohypoesthesia	N	N	N	Ambulation difficulty ptosis	leg numbness dysarthria, ataxia	N
Renal damage	RTA	N	N	dRTA diabetes insipidus	N	RTA	dRTA hematuria proteinuria	proteinuria	N	N	hematuria proteinuria
ANA	Pos	Pos	Pos	Pos	Pos	Pos	Pos	Pos	Pos	Pos	Pos
Anti-Ro/SSA	Pos	Pos	Pos	Pos	Pos	Pos	Pos	Pos	Pos	Pos	Pos
Anti-Lo/SSB	Neg	Neg	Pos	Neg	Pos	Pos	Neg	Pos	Neg	Pos	Neg
ESR	NM	NM	NM	NM	Pos	Pos	NM	Pos	Neg	Pos	NM
RF	Pos	Neg	Neg	Pos	Pos	Pos	NM	NM	Neg	NM	Neg
Hyperglobulinemic	IgG IgM	NM	NM	NM	IgG	NM	N	IgG IgA IgM	NM	NM	NM
Schirmer test	Neg	Neg	Pos	Neg	Neg	NM	Pos	Pos	Neg	NM	NM
CSF	Pos	Pos	Pos	NM	Pos	NM	NM	NM	Neg	Pos	NM
Renal biopsy	NM	NM	NM	TIN	NM	GS TIN	IgA	TIN	NM	NM	crescentic GN TIN
Salivary gland biopsy	Pos	Pos	Pos	NM	Pos	NM	NM	Pos	Pos	NM	NM
Immunomodulatory therapy	GC	GC AZA RTX	GC CTX	GC AZA MMF	GC	GC CTX MMF	GC	GC	GC	GC	GC AZA
Outcome	residual dysphagia	right-side&facial nerve weakness	vision loss	stable renal function	sensory disturbance	stable renal function	recover	stable	residual ptosis facial diplegia	recover	normal renal function
Follow up (yr)	7 m	3	0.5	6	NM	7	4 m	NM	6 m	3w	1
Case	12	13	14	15	16	17		18	19	20	21
References/Year	DeGuzman [13]	Skalova [14]	Yoshida [15]	Berman [16]	Zawadzki [17]	Kobayashi [18]		Chang [19]	Ohlsson [20]	Hoshina [21]	Present patient
Age (Gender)	14/M	16/F	13/M	10/F	15/F	10/F	11/F	15/F	8/F	16/F	12/F
Symptoms at onset	fever seizure	muscular weakness	hematuria proteinuria	fever headache dizziness vomiting	gait disturbances	uveitis	fever	weakness	arthritis	fever headache vomiting	joint pain
Dry eyes or mouth	N	N	N	NM	NM	N	N	Y	NM	Y	Y
Parotitis	N	NM	N	NM	NM	Y	Y	Y	NM	Y	N
Neurologic manifestation	muscle weakness slurred speech	muscle weakness	N	dysmetria weakness proprioception	N	N	N	N	N	N	superficial sensation impaire
Renal damage	Neg	dRTA proteinuria	hematuria proteinuria	N	dRTA	dRTA	N	dRTA	dRTA proteinuria	N	glucosuria
ANA	Neg	Pos	Pos	Pos	Pos	Pos	Pos	Pos	Pos	Pos	Pos
Anti-Ro/SSA	Neg	Pos	Pos	Pos	Pos	Pos	Pos	Pos	Pos	Pos	Neg
Anti-Lo/SSB	Neg	Pos	Pos	Pos	Pos	Neg	Pos	Neg	Pos	Pos	Pos
ESR	NM	Pos	NM	Pos	NM	Pos	Pos	Pos	Pos	NM	Pos
RF	NM	Pos	NM	Pos	NM	Pos	Pos	Pos	Pos	Pos	Neg
Hyperglobulinemic	IgG	IgG	N	IgG	IgG IgA IgM	IgG IgM	IgG	IgA	IgG1	IgG	N
Schirmer test	NM	NM	NM	NM	Neg	Pos	Neg	Pos	NM	Pos	Pos
CSF	Pos	NM	NM	Pos	NM	NM	Pos	NM	NM	Pos	Neg
Renal biopsy	NM	TIN	MGN	NM	TIN	TIN	NM	NM	NM	NM	TIN
Salivary gland biopsy	Pos	NM	Pos	Pos	Pos	Pos	NM	Neg	NM	NM	Pos
Immunomodulatory therapy	GC CTX	GC CSA	GC	GC CTX	GC CTX	GC CTX	GC	NM	GC HCQ MTX	GC TAC	HCQ GC
Outcome	lethargic visual impairment difficulty walking	recover	urine protein hematuria	stable	tubular acidosis	stable	stable	NM	synovitis	symptoms improved	glucosuria
Follow up (yr)	3	1 m	2 m	11 m	14 m	5	3	3	2 m	NM	1.5

*F female; *M* male; *Y* yes; *N* no; *m* months; *w* weeks; *NM* not mention; *Neg* negative; *Pos* positive; *GC* glucocorticoid; *AZA* azathioprine; *RTX* rituximab; *HCQ* hydroxychloroquine; *CTX* cyclophosphamide; *MMF* mycophenolate mofetil; *MTX* methotrexate; *CSA* cyclosporine A; *TAC* tacrolimus

Primary Sjogren syndrome is an autoimmune disorder that causes inflammation and injury to the exocrine glands [22], predominantly the lacrimal and salivary glands, resulting in dry eyes and mouth (sicca syndrome). There are few reports on childhood primary Sjogren syndrome, because SS is more common in adults than in children. The female to male ratio in adults is 9:1, and joint problems were present in 30–50%, while the incidence of kidney disease varies from 0.3% to up to 33.5%, depending on the study [23–27]. Other extraglandular diseases, such as cutaneous vasculitis, pulmonary manifestations, and peripheral nervous system manifestations occur in less than 10% [22]. In children, the sex ratio was 83–92.3% female [28, 29], and the most frequent symptom was parotid swelling,which was present in 42.3–53%, while central nervous system symptoms were present in 8.7%, and renal manifestations were present in 9.9–11.5%. Central nervous system and renal damage is uncommon in pediatric cases. We report a case of childhood pSS presenting with interstitial nephritis and neurological disorders.

We reviewed all full-text articles on childhood primary Sjogren syndrome and focused on cases of pediatric pSS with kidney or nerve damage (Table 1). In the review, 20/22 children were female, and 10/22 children had neurologic manifestations, often prior to the diagnosis of pSS. We reported a case in a female pediatric patient that had fever and superficial sensation disorder in the limbs months after pSS was diagnosed. Neurologic disorders included the peripheral nervous system (PNS) and the central nervous system (CNS), and involved fever, headache, mental disorder, gaze deviation, lethargy, muscle weakness, urinary incontinence, hypalgesia, difficulty with ambulation, or ptosis. Until now, no studies have been performed on the epidemiology and characteristics of neurological damage in children with pSS. The prevalence of neurologic manifestations in adults ranges between 28 and 67.5% according to the different study populations and the definition or methods for the detection of neuropathy [30–32]. The studies showed that 46–80% of adult patients with neurological symptoms developed such symptoms prior to the diagnosis of pSS [30, 31]. The frequency of constitutional symptoms and lung involvement was significantly higher in pSS with neurological involvement than in pSS without neurological involvement, and the articular symptoms were significantly less frequent in pSS with neurological involvement [33]. At present, the pathogenic mechanisms for most forms of neurological involvement in pSS have not been elucidated. However, several mechanisms have been considered to explain this involvement. In the development of PNS involvement in pSS patients, vascular or peripheral inflammatory infiltrates with or without necrosis may be found, and vasculitis of the vasa nervorum has also been proposed as a pathogenic mechanism [34–38]. These CNS disorders may be explained by three pathogenic factors. The first hypothesis involves the direct infiltration of the CNS by mononuclear cells [39]. The second hypothesis involves vascular injury that may be related to the presence of antineuronal antibodies and anti-Ro antibodies [40]. Finally, researchers [41–43] suggest that the underlying mechanism of CNS lesion development in pSS involves ischemia secondary to small vessel vasculitis.

In renal manifestations, the main damage in childhood pSS is tubulointerstitial nephritis (TIN), and the clinical features of renal involvement are proteinuria, hematuria, glucoseuria, or alkalineurine. A literature review of pediatric pSS showed that renal manifestations were present in 9.9%, including renal tubular acidosis (RTA), membranous glomerulonephritis (MGN), hypokalemia, and interstitial nephritis [28]. Glomerular damage is rare, and we only found three cases,one report a case of pSS with mesangial proliferative glomerulonephritis and IgA deposits [8], another report a girl with pSS complicated by MGN [15], the last one reported a case of pauci-immune crescentic glomerulonephritis [12]. It has been proposed that the course of renal impairment might be shorter in the interstitial nephritis group, and the incidence of cryoglobulinemia and low C4 might be higher in the glomerulonephritis (GN) group [9]. The prevalence of renal involvement in adult pSS has been reported to range from 1% in some large retrospective registries, to 5–14% in most European studies, and > 30% in a cohort of 573 Chinese patients [44]. Unlike children, the renal damage in adults is manifested by overt or latent distal RTA, nephrogenic diabetes insipidus, and rarely, proximal RTA [27, 44, 45]. The frequency of GN in adults varies from 16.6 to 58.2% depending on race, center, and number of cases [23, 24, 46–48]. In renal biopsies of GN, membranoproliferative glomerulonephritis (MPGN) secondary to cryoglobulinaemia is the most common type, and other pathological types include membranous nephropathy, IgA nephropathy, focal segmental glomerulosclerosis, minimal change disease, unspecified proliferative glomerulonephritis, crescentic glomerulonephritis, and global glomerulosclerosis [44]. A large cohort of adult pSS patients revealed that patients with TIN have a better prognosis and clinical outcome, while patients with GN have a less favorable prognosis and a higher risk of developing lymphoma, which is related to cryoglobulinemia .

From the review, except the girl we reported, 20 of 21 children were positive for ANA and anti-SSA, while, 8 out of 20 were anti-SSB negative. This result is consistent with previous observations that the positive rate of ANA and anti-SSA is higher than anti-SSB in adults and children [32, 49, 50]. From a systematic review of primary Sjogren syndrome in pediatric populations, the presence of anti-SSA (Ro), anti-SSB (La), and anti-nuclear antibodies (ANA) was positive in 36.4–84.6%, 27.3–65.4%, and 63.6–96.2%, respectively [50]. New research showed that patients positive for both anti-SSA and anti-SSB had a substantially higher risk of cerebral infarction and venous thromboembolism than the general population [51].

Meanwhile, we also found an interesting phenomenon, hyperglobulinemia presented in 12 cases, and 11/12 were IgG, only 3 cases were IgA. High IgG levels are a common feature of SS and might reflect greater B cell activation [52]. Research showed that patients with high IgG levels had a higher prevalence of purpura and immunological markers (ANA, RF, anti-SSA, and anti-SSB) [53]. All of the 3 cases with high IgA had renal damage, so we speculated whether IgA participated the pSS and kidney disease at the same time. Plasma cells producing IgA rather than IgG or IgM are dominantly observed in the salivary glands of SS patients [54]. Enrichment of anti-SS-B IgA antibodies in the saliva of patients with SS may suggest enhanced local synthesis of anti-SS-B IgA [55]. The presence of IgA autoantibodies against M3 muscarinic acetylcholine receptors is also considered to be a pathophysiological factor of primary SS [56]. These findings suggest that abnormal immunity of IgA production may attributable to SS, but unfortunately immunofluorescent findings of the 3 children indicated no deposition of IgA. More extensive investigations will be needed to support the hypothesis.

All the patients in our report received immunomodulatory therapy including glucocorticoid, azathioprine, rituximab, hydroxychloroquine, cyclophosphamide, mycophenolate mofetil, and methotrexate. At present, the treatment of childhood pSS presenting with renal and nerve damage lacks large-scale evidence-based medicine and is mostly based on clinical experience. Although the long-term outcome of renal damage in pediatric pSS is usually good, glucocorticoids were often used in pediatric cases with RTA and TIN, and chronic immunosuppression is needed for the glomerular involvement often associated with a progressive course [5, 7, 57]. Long term outcomes may be related to the improvement in overall survival rate, renal survival rate, and complete remission rate of renal disease in childhood pSS treated with immunosuppressive therapy. From our review, steroids (1 mg/kg/day) were often used as the initial immunosuppressive treatment in pediatric pSS patients with TIN or GN. However, the outcome of children presenting with neurological diseases in the review is relatively less favorable. All the cases here reported residual neurologic manifestations, even though these children received steroids (1–2 mg/kg/day) or other immunosuppressive therapies. Kornitze [3] reviewed reported cases of childhood Sjogren syndrome with CNS complications in which 10 of 10 cases received steroid therapy and 7 of 10 patients had residual neurological deficits at follow-up. The effectiveness of glucocorticoids combined with other immunosuppressants in children still requires further study.

The frequency of renal damage and neurologic involvement in childhood pSS is much lower than in adults, and the clinical symptoms may be significantly different than what is seen in adults. If children present with unexplained renal diseases, neurological abnormalities, and unexplained fever, pSS should be considered. Special serological screening tests should be added to routine laboratory tests, while kidney biopsy may contribute to assessing the extent of renal damage and the need for immunomodulatory therapy. Early recognition and the appropriate treatment of renal damage and neurologic involvement would improve prognosis and prevent complications. Long term follow-up is essential for these children due to the possibility of rapid progression.

## Data Availability

Data sharing not applicable to this article as no datasets were generated or analyzed during the current study.

## Abbreviations

SS: Sjögren syndrome
pSS: primary Sjogren syndrome
EGMs: extraglandular manifestations
ESR: erythrocyte sedimentation rate
OGTT: oral glucose tolerance test
HbA1c: glycosylated hemoglobin A-1c
RG: renal glucosuria
ANA: anti-nuclear antibody
anti-SSB: anti-Sjögren syndrome B
P-ANCA: perinuclear antineutrophil cytoplasmic antibody
anti-SSA: anti-Sjögren syndrome A
anti-dsDNA: anti-double stranded DNA
anti-RNP: anti-ribonnucleoprotein
anti-MPO-ANCA: anti-myeloperoxidase antineutrophil cytoplasmic antibody
RF: rheumatoid factor
anti-CCP: anti-cyclic citrullinated peptide
HBV: hepatitis B virus
HCV: hepatitis C virus
HIV: human immunodeficiency virus
CSF: cerebrospinal fluid
PNS: peripheral nervous system
CNS: central nervous system
TIN: tubulointerstitial nephritis
RTA: renal tubular acidosis
GN: glomerulonephritis
MGN: membranous glomerulonephritis
MPGN: membranoproliferative glomerulonephritis
